# COVID‐19's impact on type 1 diabetes management: A mixed‐methods study exploring the Peruvian experience

**DOI:** 10.1002/hpm.3536

**Published:** 2022-07-05

**Authors:** Jessica Hanae Zafra‐Tanaka, Lizzete Najarro, Janeth Tenorio‐Mucha, Maria Lazo‐Porras, Diego Bartra, Gina Bazán, María Claudia Berghusen Anteparra, Ursula Bonilla, Vicky Motta, David Beran

**Affiliations:** ^1^ CRONICAS Centre of Excellence in Chronic Diseases Universidad Peruana Cayetano Heredia Lima Peru; ^2^ Division of Tropical and Humanitarian Medicine University of Geneva and Geneva University Hospitals Geneva Switzerland; ^3^ Asociación de Diabetes en Niños y Adolescentes (ADINA Perú) Lima Peru; ^4^ Diabetes 1 Perú (DM1 Perú) Lima Peru; ^5^ Asociación de Diabetes Juvenil del Perú (ADJ) Lima Peru

**Keywords:** COVID‐19, health care system response, type 1 diabetes mellitus

## Abstract

**Introduction:**

The COVID‐19 pandemic has negatively impacted health services, especially in low‐and‐middle‐income countries, where care for chronic conditions such as diabetes was disrupted. Our study aims to describe the challenges faced by people living with Type 1 diabetes mellitus (T1DM) to access care during the COVID‐19 pandemic in Peru.

**Methods:**

A sequential explanatory mixed‐method study was conducted between May and September 2020 including health professionals involved in T1DM care, people with T1DM and their caregivers. The study consisted of a quantitative strand to gather general information through electronic surveys and a qualitative strand that involved in‐depth interviews.

**Results:**

For the quantitative study, we included 105 people with T1DM, 50 caregivers and 76 health professionals. The qualitative study included a total of 31 interviews; 16 people with T1DM, 14 health care professionals, and one representative from the Peruvian Ministry of Health (MoH). People with T1DM faced difficulties accessing consultations, insulin, monitoring devices and laboratory testing during the pandemic. Different phases of the Peruvian health system response were found. Firstly, an initial informal response to addressing T1DM care during the pandemic characterised by local initiatives to ensure continuity of care for people with T1DM. Following from this, a formal response was implemented by the MoH which focussed on reinforcing the primary level of care. Measures included teleconsultations and delivery of medicines, although these were not implemented in all health care establishments. Throughout the pandemic patient associations played an important role in organising and helping to counteract the impact of COVID‐19 on people with T1DM.

**Conclusions:**

The Peruvian health care system slowly adapted to the COVID‐19 pandemic to provide care for people with T1DM. However, people with T1DM had difficulties to access care. Thus, reinforcement of interventions such as communication between levels of care, teleconsultations and delivery of medicines was urgently needed. Patient associations' capacity to respond should be considered by local authorities and civil society should be part of the health system response.

## Highlights

- In Peru, non‐communicable diseases (NCD) care was initially neglected during the pandemic given the scarcity of resources.
- People with Type 1 diabetes mellitus (T1DM) had limited access to consultations and insulin.
- Civil society organised and mitigated the impact of COVID‐19 on health care access.
- Civil society should be involved by local authorities when planning for public health interventions.

## 1 INTRODUCTION

COVID‐19 generated a huge direct and indirect impact on people living with non‐communicable diseases (NCD) such as type 1 (T1DM) and type 2 diabetes (T2DM).^1^, ^2^ People with diabetes faced more severe cases and higher mortality rates due to SARS‐CoV2. Additionally, people with diabetes had limited access to healthcare services^3^ due to allocation of resources to COVID‐19 management,^4^ lock down measures, and negative impact on household finances given reductions in salaries or loss of jobs. These circumstances resulted in difficulties accessing treatment as well as healthy food.^5^, ^6^, ^7^, ^8^

People with T1DM were particularly vulnerable given that they needed continued access to medical care, insulin and monitoring devices. Access to care for people with diabetes varied when contrasting different country settings. In low‐and‐middle‐income countries (LMIC),^9^, ^10^ it was reported that people with diabetes had difficulties in accessing care. In contrast studies from high‐income countries (HIC) showed that people with T1DM had good management of their disease during lockdown.^9^

In Peru the first case of SARS‐COV‐2 was identified in March 2020 and on the 11^th^ of March, the government declared a state of emergency that was prolonged until March 2021.^11^ The pandemic intensified the fragmentation and inequalities within the Peruvian health care system.^12^ As a consequence, the government faced difficulties in providing an effective response to the pandemic and Peru had one of the highest excess mortality rates worldwide.^13^ In May 2020, the Ministry of Health [MoH] launched an initiative to reinforce primary health care and prioritised different services including follow‐up of NCDs such as diabetes.^14^ As of July 2021, the health system had adapted in different ways: coordination of activities and care provision between the different subsystems (MoH, Social Security, Armed forces and private sector), increasing the number of critically‐ill unit beds and ventilators, trying to provide enough oxygen supply and reducing the number of clinical consultations, among others.

Our study aimed to describe the Peruvian health care system's response to managing T1DM and to describe the challenges faced by people with T1DM to access diabetes care and insulin during the COVID‐19 pandemic in Peru.

## 2 METHODS

### 2.1 Study design

A sequential explanatory mixed‐method study, consisting of a quantitative strand conducted between May and August 2020 which aimed to gather general information through electronic surveys and a qualitative strand conducted between September to November 2020 which involved in‐depth interviews. Interviewees included health care professionals involved in T1DM care, and people with T1DM or their caregivers. The study was conducted in Peru and aimed to collect data nationwide.

The results from the quantitative strand informed the development of the interview guides for the qualitative component and the selection of participants.^15^ As an example, interview guides were modified for an in‐depth exploration the most relevant issues found when analysing the online surveys. In the case of selection, a disproportionate number of people treated in private clinics answered the online survey. Thus, we decided to sample more people treated at the MoH and Social security establishments in the qualitative study to complement the information collected in the online surveys.

This study is part of the Addressing the Challenge and Constraints of Insulin Sources and Supply (ACCISS) study,^16^ which aims to improve access to insulin. In‐country activities tailored to the countries' needs are currently being conducted in Kyrgyzstan, Mali, Peru and Tanzania. This study is part of the activities conducted in Peru^17^ with the aim to better understand the impact of SARS‐COV‐2 on access to care for people with Type 1 diabetes.

This study was conducted by Peruvian researchers who developed the online surveys and interview guides in Spanish, conducted the interviews in Spanish, and transcribed and analysed the data. Finally, the researchers translated quotations into English to be included in the manuscript.

### 2.2 Context

Before the pandemic, the Peruvian health care system had several weaknesses concerning NCD care, including: different health care providers (MoH, Social Security, Armed forces and private clinics) with little coordination between them; a lack of strong health information systems that prevented knowing the exact numbers of people with DM; and limited resources (human, infrastructure). These weaknesses created high patient out‐of‐pocket expenses, problems to access medicines, laboratory check‐ups and follow‐up appointments.^18^

It is estimated that around 3860 people live with T1DM in Peru.^19^ Before the pandemic, they received centralised care in hospitals mainly located in the capital of the country. Additionally, primary health care was not adapted for T1DM care. Both situations forced people with this condition to travel to the capital city to access medicines, laboratory testing and follow‐up visits.

### 2.3 Participants

We included (1) health care professionals (endocrinologists, general physicians, nurses, and pharmacists) who provided care to people with T1DM, and 2) people living with T1DM or their caregivers (if the person was under 18 years old) who receive care from MoH, Social security and private sector in Lima and provinces. We excluded potential participants that could not communicate due to language barriers, did not provide their consent to participate in the study or were not willing to participate.

For the quantitative strand, we conducted online surveys using REDCap and disseminated them through emails and social media (Facebook, Twitter and WhatsApp) with the help of patients' organizations (Asociación de Diabetes Juvenil [ADJ],^20^ Asociación Diabetes 1 Perú [DM1 Perú] y Asociación de Diabetes en Niños y Adolescentes [ADINA Perú]^21^), endocrinology and pediatric societies, and colleagues with whom we have previously worked.

For the qualitative strand, we invited participants from the quantitative study who expressed interest in participating in the in‐depth interviews and, when we had exhausted that list, we sought participants through patient associations, contacts among health care providers and people we had collaborated with in the past. Participants were selected following a theoretical sampling (see Table 1) and the sample size was defined based on the theoretical saturation. The interviews were conducted via telephone calls by a qualitative researcher. The interviews were recorded and transcribed verbatim.

**TABLE 1:** Characteristics of the participants

Characteristics	Patients and caregivers (*N* = 155) n (%)	Health care professionals (*N* = 76) n (%)
Age in years	25 (16–36)^a^	42.3 +‐ 14.8^b^
Gender		
Female	85 (54.8)	40 (52.6)
Male	60 (38.7)	27 (35.5)
Prefer not to answer	0 (0.0)	2 (2.6)
Missing	10 (6.5)	7 (9.2)
Diabetes educator		
Is an educator	NA	48 (63.1)
Is not an educator	NA	20 (26.3)
Missing	NA	8 (10.5)
Region		
Other regions	17 (11.0)	29 (38.2)
Lima	119 (76.8)	35 (46.1)
Missing	19 (12.3)	12 (15.8)
Health care system^c^		
MoH (MINSA)	16 (10.3)	38 (50.0)
Social security (EsSalud)	50 (32.3)	13 (17.1)
Arm forces	1 (0.6)	4 (5.3)
Private	54 (34.8)	14 (18.4)
No insurance	16 (10.3)	NA
Missing	23 (14.8)	7 (9.2)
Level of care		
Health center (primary level of care)	NA	8 (10.5)
Hospital (tertiary level of care)	NA	40 (52.6)
Specialized institute	NA	6 (7.9)
Missing	NA	22 (28.9)
Profession		
Family doctor	NA	12 (15.8)
Endocrinologist	NA	34 (44.7)
Pediatric endocrinologist	NA	6 (7.9)
Pediatrician	NA	1 (1.3)
Nurse	NA	7 (9.2)
Nutritionist	NA	7 (9.2)
Missing	NA	9 (11.8)

*Note*: Not applicable (NA) Some participants did not answer all the questions, we then provide the number of records with missing information for each variable.

^a^ median (IQR).

^b^ mean + ‐ SD.

^c^ for patients we present insurance.

Theoretical sampling refers to a form of purposive sampling that allows researchers to select new cases based on their potential to extend or test the understanding of the subject being studied.^22^, ^23^ We selected this method as it is concept‐based rather than participant‐based, this means that the participants were selected based on the information they could provide regarding the themes/categories that emerged from the initial interviews.^24^ Theoretical saturation refers to an iterative process of data collection and analysis whereby data collection is governed by emerging theory rather than predefined characteristics of the population.^25^

### 2.4 Ethics statement

Our study was approved by the Universidad Peruana Cayetano Heredia Institutional Review Board and patient confidentiality was protected.

### 2.5 Data analysis

For the quantitative strand, we conducted data cleaning and assessed the consistency of data using Stata v15 software. We used absolute and relative frequencies to summarise categorical variables and central tendency and dispersion measures for numerical variables according to the distribution.

For the qualitative strand, themes, categories and codes were identified after reading a small selection of interviews. This information was used to build an initial version of the codebook. Then, each of the interviews was analysed using the software ATLAS.ti.^26^ The qualitative researcher sought similarities and differences between the participants' experiences. The findings were discussed with the principal investigators as the analysis was being conducted to clarify specialised concepts (e.g., medical terms). To integrate quantitative and qualitative data at the interpretation and reporting level, we have used a narrative approach and we are presenting the results jointly in tables.^27^

## 3 RESULTS

We present the results in three sections: (1) characteristics of study participants, (2) a description of the health system response and adaptation in phases, and (3) access to insulin during this period (See Supplementary Tables for more details).

### 3.1 Characteristics of the participants

A total of 212 participants completed the survey for people living with T1DM or caregivers. Of them, 57 did not meet inclusion criteria or did not provide informed consent. Thus, the final sample included a total of 105 people with T1DM and 50 caregivers. Both caregivers and patients provided information about people with T1DM. Thus, we will present answers from both types of informants aggregated. The median age of the people with type 1 diabetes was 25 years (IQR: 16–36) and most (85/155) were females. A majority of them (119/155) lived in Lima, the capital city, with the remainder in 10 other regions of Peru.

A total of 182 participants filled out the survey for health professionals. Of them, 106 did not meet inclusion criteria or did not provide informed consent. Thus, of 76 health professionals are included in our analysis. The mean age of the health professionals was 42.3 years (SD: 14.8) and most (40/76) were females. Half of the participants worked in Lima (35/76) and the rest of them lived in different regions (See Table 1).

For the qualitative study, a total of 31 interviews were included. This comprised: 16 people with T1DM with ages ranging from 6 to 68 (in the case of those younger than 18 years, we interviewed their main caregiver); 10 health care professionals including endocrinologists, pediatric endocrinologists and nutritionists, 4 pharmacists; and one representative from the MoH (Supplementary Table 1).

### 3.2 Health system response and adaptation

#### 3.2.1 Reacting to the emergency and the system's informal response

Within the public health measures implemented as of the 16^th^ of March 2020, there was the cancelation of outpatient care services for all public and private health facilities which meant no clinical visits, no laboratory and no public pharmacies were open (See Supplementary Table 2).

There was a reduction in the human resource capacity for diabetes care as a consequence of different measures that were implemented. One of these measures was the creation of special emergency, hospitalisation and laboratory services to respond to the COVID‐19 pandemic resulting in human resources being diverted from other areas of care. The other challenge was that many health professionals in the Peruvian health system have contracts which are renewed every 3 months and the budget for non‐COVID health services care was decreased. This meant that some endocrinologists did not have their contracts renewed and went to work in COVID care. Both these factors resulted in fewer personnel being available for routine care including diabetes.

At the beginning of the pandemic and due to the lack of outpatient services, people with T1DM did not know how to access consultations and where to get their insulin prescription. Some participants who kept in contact with their physicians, could not go to the pharmacies because they were not open for outpatient care. In the case of some participants living outside the capital and who were not able to travel, they asked a family member to get the medicines carrying the patient's identification documents.

respondent: …we have always been in contact with patients through virtual means (…) The only great difficulty was with patients who live in the regions. So, we solved that by telling any family member to come and collect the insulin presenting the child's papers.(Provider_MoH_Lima)

As a first informal response, health care providers independently adapted and sought the most suitable solutions to continue treating people living with T1DM. They used different strategies such as follow‐ups using phone calls or reaching them through social media (WhatsApp or Facebook) by either personal or group channels. This was feasible given the small number of people living with T1DM and the past usage of these alternative communication strategies.

Health care professionals provided care through face‐to‐face consultations, emergency room, hospitalizations and teleconsultations. However, from the survey, most (54/76) of the professionals referred that the number of consultations for people with DM had been reduced in comparison with the month before the emergency state.

The public health emergency and the adaptation of the system also impacted participants' behaviours when seeking care. When surveyed with regard to treatment during the emergency state, 115 participants living with T1DM provided information. Of them, 65 referred to have sought medical care nearly half (33/65) did not have access to medical consultations. Among those who did not seek medical care, the main reasons were fear (30/50), considering it was not needed (9/50), hospitals not providing care for chronic patients' (9/50) and lack of money (4/50).

Lockdown created difficulties in transportation to health care facilities. Furthermore, the closure of borders between regions prevented patients who lived outside Lima but were treated there from attending their appointments and accessing their treatment. For people with T1DM, the difficulties to access clinical consultations were related to problems getting prescriptions for insulin. Moreover, the closure of external consultation pharmacies prevented access to medication. As for laboratories, their restriction to external users caused difficulties for patients to conduct tests to monitor T1DM (e.g., glycated hemoglobin).

respondent: …the laboratory (tests) that could not be done was the routine controls. That itself has been negatively impacted, glycosylated hemoglobin controls, kidney function tests, evaluations of complications. Of course, the periodic evaluations that were done in normal times have been lost… (Provider_EsSalud_Region)

#### 3.2.2 System's formal response

Four months after the start of the COVID lockdown in Peru, in mid‐July 2020, the MoH published guidelines^28^, ^29^ regulating the organization of health care for people with NCDs and the prescription and distribution of medicines and devices for these patients. The guidelines suggested the integration of care for people with NCDs depending on the level of care. For the primary level of care, the MoH suggested the following steps: (1) identification of people with NCDs using available registries at a regional level; (2) contacting people and offering them a teleconsultation at the primary health care level to assess complications of the disease and availability of medicines; and (3) coordination with the services depending on the initial assessment's findings. While, for the secondary and tertiary levels of care, the suggestion was as follows: (1) publish a contact number so patients can get remote consultations; (2) assess the patients for complications and availability of medicines; (3) in case the patients need medicine, do a prescription and coordinate with the closest health care establishment and local organizations to deliver the medicines (See Supplementary Table 3).

Remote consultations were one of the many strategies implemented to ensure follow‐ups for people with NCDs. However, healthcare providers highlight the lack of preparedness in the health care system and the need to improve telemedicine services. Some of the barriers identified were: (1) lack of an updated registry of people with NCD and difficulties to contact them; (2) lack of infrastructure, including call centers to schedule the appointments, equipment and internet services to conduct consultations; and (3) lack of trained personnel to work at call centers. This lack of personnel was present despite the MoH having conducted training for health care professionals to use the informatics platforms and to communicate the process related to patient care such as appointments, coordinating referrals, and prescription of drugs.

Remote consultations were perceived as an opportunity to preserve the continuity of care. But both patients and health care providers considered that they were not enough to fulfil patients' needs given the lack of physical examination, laboratory test results, and the difficulties in the interaction. For these reasons, professionals considered they could be used as a complement to in‐person consultations in the future.

respondent: telemedicine is complementary to face‐to‐face clinical visits. I think it (the pandemic) was an opportunity to, let's say, get started on this and continue using it. It is an opportunity that allows us to follow the patient (Providers_MoH_Lima)

Health care professionals proposed different ways to improve the remote consultation services. Firstly, this was by prioritising high risks patients (e.g. children or adolescents with T1DM with poor disease control). Next it was using remote consultations as a complement to in‐person consultations to allow more frequent consultations. The creation of a virtual platform that allowed reviewing clinical records to conduct a better follow‐up of patients, improve patient registries was another component. Training so health care personnel were familiarised with the tools was also part of this response, as was improving communication around these services as providers felt patients were not always aware of what was available to them and what steps to take to get a remote consultation.

### 3.3 Access to insulin

With the implementation of remote consultations, physicians were able to ‘extend’ the prescriptions of their patients, which meant continuing the medication they were already using, which could then be picked up at the hospital pharmacy (See Supplementary Table 4).

Despite these measures, some participants had difficulties accessing insulin because of the restrictions on movement, especially those who lived in regions other than Lima, the long queues in health facilities, and the lack of availability of insulin in some health facilities. People with T1DM and caregivers' responses to these barriers consisted of reducing the dose of insulin, replacing the insulin they were using with another one available or cheaper, coordinating with patient associations, family and friends helping get and deliver the insulin, and buying insulin.

respondent:…they only gave me an NPH, the R I had to buy.

interviewer: Do you still have the medications?

respondent: No, not anymore.

interviewer: And how do you do then?

respondent: I have a friend who also has diabetes and… sometimes he gives me his insulin (Patient_MoH_Lima)

Due to the barriers to access to insulin, patient associations played a crucial role. They provided an important social support network that allowed sharing of information, medicines and devices. As an example, some members donated medicines they received from health insurance and did not use to members who could not access them. This allowed patients who had no access to medicines and devices, especially those who lived in other regions, to access medication. Additionally, even during the public health emergency, patient associations continued conducting different activities that allowed the exchange of information and peer support, such as online meetings.

In Peru, some people decided to buy insulin because they wanted to use a specific type of insulin that was not usually provided by their insurance. During the pandemic, some participants bought the insulin at a private pharmacy rather than attending a health facility because of the fear of getting infected with COVID‐19. Additionally, some had to pay for the delivery of insulin from one region to another one given that not all insulins were available in the regions and due to the restrictions to travel to other regions. However, the average expenditure on insulin remained stable (200 PEN [IQR: 150–300] and 250 [IQR: 150–360], pre and during COVID‐19 pandemicrespectively, *p*‐value = 0.375).

The system's response to the lack of availability of insulin included changing the prescription and coordinating the transfer of insulin from public pharmacies with low usage of insulin to those with higher usage of insulin.

Overall, the public health emergency combined with the challenges in accessing insulin also impacted mental health as patients and caregivers reported feeling anxious or sad. In addition, having T1DM, made them feel more vulnerable than the rest of the population.

respondent:…it would be very important to measure how (the pandemic) has affected mental health of children with diabetes and the family (…) I live it, because my son is very active, he plays soccer, swimming. Right now, he's locked up, I'm afraid to take him out because he's vulnerable. What he does is (…) Sometimes he tells me 'oh, mom, I don't like diabetes, I hate diabetes.' (Patient_association)

Nutrition and physical activity were also affected by the COVID‐19 lockdown. As an example, participants referred to difficulties following an adequate diet in the first months of the lockdown. This was mainly due to transport restrictions which generated food shortages and a rise in prices. This was particularly important for fruits and vegetables that are usually produced in some regions of the country and redistributed by trucks to other regions.

As for physical activity, the lockdown forced people to stay home which led to a reduction in exercise. Additionally, for some people, exercise was part of school activities. Thus, with classes being cancelled, their motivation to be physically active disappeared.

## 4 DISCUSSION

The COVID‐19 pandemic in Peru had a significant impact on T1DM management. Because of a strict national lockdown, closure of healthcare services and financial difficulties, patients struggled to access clinical appointments, laboratory testing, insulin, and blood glucose monitoring supplies. The health system's response can be seen in two distinct phases: the first phase is characterised by local initiatives to continue care for patients, and the latter is led by the MoH which aimed to organise care for NCDs reinforcing the primary level of care. Measures included teleconsultations and delivery of medicines. However, these were not implemented at all healthcare establishments. Even when the health system tried to adapt and respond to the needs of people with T1DM, this answer was delayed and had many challenges in its implementation due to limited resources causing negative effects on the treatment of this population (See supplementary Table 5 for a summary).

The onset of COVID‐19 caused health systems to focus their attention on this disease neglecting care for patients with other conditions to different degrees around the world.^30^ This included health staff being reassigned to COVID‐19 efforts, funds allocated to NCDs being reallocated to COVID‐19 in 20% of the countries, and limited access for outpatient services for NCDs in 55% of countries worldwide.^31^ During the first phase of the response in Peru, outpatient services were completely closed. This led to a lack of access to follow‐up visits and medicines for people with T1DM. During the second phase, outpatient services progressively started to work with restrictions and some measures were implemented to mitigate the impact of COVID‐19 on NCD care including T1DM with telemedicine.

The pandemic caused telemedicine (remote consultations through call or video calls) to become the principal way to receive care. Even when this has several advantages, some challenges were reported by healthcare providers, such as: lack of time and staff; limited knowledge on how to use technological tools; and problems contacting patients due to outdated contact information.^3^, ^10^ Future actions for clinicians and health care systems should include identifying and preventing potential disparities in telemedicine access, tackling barriers to digital literacy, and improving financing and infrastructure to provide care in a remote way.^32^ Also, there is a need to ensure privacy and confidentiality, advocate for an expansion of high‐speed internet access to rural and underserved communities, and encourage the continuation of telehealth.^33^

Telemedicine as a response to T1DM needs was perceived as insufficient as the scarce time allocated for consultations was focussed on assessing whether the patients had insulin. However, education on how to use insulin, how to eat and how to exercise could not be properly delivered. Moreover, during the pandemic, support on how to adapt nutrition and physical activity to the numerous restrictions faced during lockdown was much needed. As an example, nutrition was affected due to the reduction of family income, either because of the loss of jobs or reallocation of the family budget to buy alcohol and masks to fight against COVID‐19, and limited access to markets. These changes in nutrition coupled with sedentarism, which increased due to the restrictions to go out, complicated control of diabetes. Thus, management should be comprehensive and include on‐going education on self‐care behaviours such as: eating healthy, being active, monitoring, taking medication, problem solving, healthy coping and reducing risk, as proposed by the American Association of Diabetes Educators.^34^ This comprehensive management could be achieved using a hybrid‐approach of telemedicine and in‐person visits including individual consultations and group education sessions.

Specifically, people with T1DM faced challenges accessing insulin and glucose monitoring devices during curfews and lockdowns. A worldwide survey stated that almost one‐quarter of healthcare providers perceived that COVID‐19 caused a shortage in glucose test strips and insulin.^35^ Whereas information collected from people estimated that 8% and 6% had difficulties getting their insulin and continuous glucose monitors, respectively.^36^ Some interventions were successfully implemented to address this. For example, in Australia when stockpiling and localised shortages of medicines and devices occurred, the government established a delivery limit for diabetes products and educated the population to keep their necessary supplies and not stockpile to not affect others.^37^ In India, it was proposed to circulate the contact numbers of suppliers and support programs to improve access to essential care products and services for diabetes.^38^ Given the importance of insulin and glucose monitoring devices for the management of T1DM, specific strategies need to be implemented to secure these vital medicines and devices.

Patient organizations are important allies in diabetes care, they contribute to patients' psychosocial support, education, and prevention of complications; besides, they can play a mediator role between health and social systems.^39^ All of those actions can be helpful during COVID‐19 since patients experience a lack of access to formal diabetes education and some of them need social assistance for food or medicines. In Australia, patient organizations made information available regarding T1DM management during COVID‐19, triage for diabetic foot, symptoms of T1DM for early diagnosis, and telehealth options through websites, Facebook and Twitter, but also organising webinars, webchats, and podcasts.^37^ In Peru, patient associations worked on providing information and support to patients and their caregivers.

Regarding education and training of healthcare providers, the MoH focussed on T2DM management and did not train the healthcare personnel at the first level of care to manage T1DM. This was particularly deleterious for the care of people living with T1DM in Peru, as treatment is usually provided in hospitals that were not providing consultations during the pandemic. Thus, reinforcing the primary level of care to provide services to people living with chronic conditions without training the personnel on T1DM, left a gap in the care of these patients. In the future, coordination between different actors including the MoH, patient associations and the education system (e.g., the Peruvian School of Public Health [ENSAP] or universities) should be prioritised to provide continuous training and accurate information on the management of T1DM. Dissemination of this information could be facilitated using Information and communications technology (ICTs) or social media to reach specific audiences such as healthcare personnel, but also patients and their caregivers and school teachers who are in touch with these patients on a day‐to‐day basis.

The COVID‐19 pandemic forced the health care system to adapt and many of the interventions implemented to secure continuity of care of NCDs such as teleconsultation and telemonitoring, delivery of drugs at patients' homes or nearby pharmacies and decentralised laboratory testing strategies can potentially be reinforced and use even after the pandemic is over. All of these strategies rely on an updated registry that can help identify the patient's diagnostic, treatment, telephone contact and address, and on a strong primary level of care that needs to work in coordination with hospitals' laboratories and pharmacies and act as a mediator to bring those services to the patient's homes. As the above‐mentioned interventions can be used to improve health services after the pandemic is over, we recommend strengthening the information systems to provide timely and accurate information on patients with NDCs to tailor interventions and assess their impact.

### 4.1 Strengths and limitations

We present the results of a mixed‐methods study with a sequential explanatory design that allowed us to quantify and to better understand the barriers faced by people with T1DM when trying to access care during the COVID‐19 pandemic and the health systems response. This study is the effort of different stakeholders that provided relevant perspectives when interpreting the results and providing recommendations for the health care system.

However, our study has some limitations. For the quantitative strand, selection bias due to online survey usage and their dissemination through social media was present. This limits the reach of our study to those who have internet access and/or were linked to or followed a patient association. We also found that most of the patients and caregivers had either private or social security insurance that only covers respectively 6% and 29% of the population in the country^40^: People accessing these schemes have better socioeconomic status. However, when conducting the qualitative strand, we included participants living in the capital city and regions who had different types of insurance. This allowed us to explore the different subsystems' responses in different parts of the country. Finally, not all participants provided an answer to all the survey questions. We have then reported when information in missing.

Additionally, we invited people living with T1DM or their caregivers to participate in the study. Thus, those who self‐identified as having T1DM participated and we did not confirm the T1DM diagnosis through testing. To minimise this selection bias, we included questions related to age at diagnosis and prescribed treatment. These questions allowed us to have some certainty that patients had T1DM as we expect an early onset in contrast with T2DM patients and insulin as the main treatment.

## 5 CONCLUSION

The COVID‐19 pandemic heavily impacted the Peruvian health care system which adapted care for T1DM in two different phases: the first one characterised by local initiatives (or informal responses) to continue care for patients, and the latter led by the MoH which aimed to organise care NCDs reinforcing the primary level of care. In the case of people with T1DM, difficulties to access consultations, insulin, monitoring devices and laboratory testing were found. The MoH response consisted mainly of teleconsultations and delivery of medicines that did not reach all patients and were implemented late. However, patient associations managed to organise and help counteract the impact of COVID‐19 on patients with T1DM health. Many of the interventions implemented during the COVID‐19 pandemic could be reinforced and included within the regular health care services even after the pandemic.

The findings of this study highlighted the necessity to prepare the health system to properly answer patients' needs in general and especially during emergencies. The health system has to be organised from the beginning and be led by the MoH to protect everyone, especially those who are most vulnerable.

## CONFLICT OF INTEREST

The authors declare no conflict of interest.

## ETHICS STATEMENT

Our study was approved by the Universidad Peruana Cayetano Heredia Institutional Review Board and patient confidentiality was protected.

## Supporting information

Supplementary Material

Supplementary Material

## Data Availability

The data that support the findings of this study are available on request from the corresponding author. The data are not publicly available due to privacy or ethical restrictions.

## References

[1] Huang I , Lim MA , Pranata R . Diabetes mellitus is associated with increased mortality and severity of disease in COVID‐19 pneumonia ‐ a systematic review, meta‐analysis, and meta‐regression. Diabetes Metabol Syndr. 2020;14(4):395‐403. 10.1016/j.dsx.2020.04.018 PMC7162793 32334395

[2] Barron E , Bakhai C , Kar P , et al. Associations of type 1 and type 2 diabetes with COVID‐19‐related mortality in England: a whole‐population study. lancet Diabetes & Endocrinol. 2020;8(10):813‐822. 32798472 10.1016/S2213-8587(20)30272-2 PMC7426088

[3] Danhieux K , Buffel V , Pairon A , et al. The impact of COVID‐19 on chronic care according to providers: a qualitative study among primary care practices in Belgium. BMC Fam Pract. 2020;21(1):255. 10.1186/s12875-020-01326-3 33278877 PMC7718831

[4] Centers for Disease Control and Prevention (CDC) ‐ National Center for Health Statistics . Reduced Access to Care; 2020. Accessed April 27th, 2021. https://www.cdc.gov/nchs/covid19/rands/reduced‐access‐to‐care.htm#limitations

[5] Saqib MAN , Siddiqui S , Qasim M , et al. Effect of COVID‐19 lockdown on patients with chronic diseases. Diabetes Metabol Syndr. 2020;14(6):1621‐1623. 10.1016/j.dsx.2020.08.028 PMC7450263 32889403

[6] Chudasama YV , Gillies CL , Zaccardi F , et al. Impact of COVID‐19 on routine care for chronic diseases: a global survey of views from healthcare professionals. Diabetes Metabol Syndr. 2020;14(5):965‐967. 10.1016/j.dsx.2020.06.042 PMC7308780 32604016

[7] Posel D , Oyenubi A , Kollamparambil U . Job loss and mental health during the COVID‐19 lockdown: evidence from South Africa. PLOS One. 2021;16(3):e0249352. 10.1371/journal.pone.0249352 33784339 PMC8009396

[8] Griffiths D , Sheehan L , van Vreden C , et al. The impact of work loss on mental and physical health during the COVID‐19 pandemic: baseline findings from a prospective cohort study. J Occup Rehabil. 2021. 10.1007/s10926-021-09958-7 PMC7926060 33656699

[9] Aragona M , Rodia C , Bertolotto A , et al. Type 1 diabetes and COVID‐19: the “lockdown effect”. Diabetes Res Clin Pract. 2020;170:108468. 10.1016/j.diabres.2020.108468 32987040 PMC7518840

[10] Matheus ASM , Cabizuca CA , Tannus LRM , et al. Telemonitoring type 1 diabetes patients during the COVID‐19 pandemic in Brazil: was it useful? Arch Endocrinol Metabol. 2020. 10.20945/2359-3997000000309 PMC10528697 33166438

[11] Peruano G . Declaratoria de Emergencia Sanitaria Nacional. Gobierno Peruano; 2020.

[12] Gianella C , Iguiñiz‐Romero R , Romero MJ , Gideon J . Good health indicators are not enough: lessons from COVID‐19 in Peru. Health Hum. Right. 2020;22(2):317‐319. PMC7762920 33390718

[13] Taylor L . Covid‐19: why Peru suffers from one of the highest excess death rates in the world. BMJ. 2021;372:n611. 10.1136/bmj.n611 33687923

[14] Ministerio de Salud (MINSA) . Nts N°160‐Minsa/2020. Norma Técnica De Salud Para La Adecuación De La Organización De Los Servicios De Salud Con Énfasis En El Primer Nivel De Atención De Salud Frente A La Pandemia Por Covid‐19 En El Perú. Peru; 2020.

[15] Creswell JW , Clark VLP . Choosing a Mixed Methods Research Design. Designing and Conducting Mixed Methods Research. Sage publications; 2017.

[16] Health Action International (HAI) . About ACCISS; 2022. Accessed March 29th, 2022. https://haiweb.org/what‐we‐do/about‐acciss/

[17] CRONICAS Centro de Excelencia en Enfermedades Cronicas . Acciones para mejorar el acceso a Insulina en el Perú – ACCISS Fase 2; 2022. Accessed March 29, 2022. https://cronicas‐upch.pe/acciones‐para‐mejorar‐el‐acceso‐a‐insulina‐en‐el‐peru‐acciss‐fase‐2/

[18] Cardenas MK , Miranda JJ , Beran D . Delivery of Type 2 diabetes care in low‐ and middle‐income countries: lessons from Lima, Peru. Diabet Med J Br Diabet. Assoc. 2016;33(6):752‐760. 10.1111/dme.13099 27194174

[19] Green A , Hede SM , Patterson CC , et al. Type 1 diabetes in 2017: global estimates of incident and prevalent cases in children and adults. Diabetologia. 2021;64(12):2741‐2750. 34599655 10.1007/s00125-021-05571-8 PMC8563635

[20] Boko‐Collins PM , Ogouyemi‐Hounto A , Adjinacou‐Badou EG , et al. Assessment of treatment impact on lymphatic filariasis in 13 districts of Benin: progress toward elimination in nine districts despite persistence of transmission in some areas. Parasites Vectors. 2019;12(1):276. 10.1186/s13071-019-3525-5 31146779 PMC6543600

[21] Adinan J , Manongi R , Temu GA , et al. Preparedness of health facilities in managing hypertension & diabetes mellitus in Kilimanjaro, Tanzania: a cross sectional study. BMC Health Serv Res. 2019;19(1):537. 10.1186/s12913-019-4316-6 31366384 PMC6670222

[22] Hancock B , Ockleford E , Windridge K . An Introduction to Qualitative Research. Trent focus group; 2001.

[23] Moser A , Korstjens I . Series: practical guidance to qualitative research. Part 3: sampling, data collection and analysis. Eur J Gen Pract. 2018;24(1):9‐18. 29199486 10.1080/13814788.2017.1375091 PMC5774281

[24] Wuetherick B . Basics of qualitative research: techniques and procedures for developing grounded theory. Can J Univ Cont Educ. 2010;36(2). 10.21225/d5g01t

[25] Vasileiou K , Barnett J , Thorpe S , Young T . Characterising and justifying sample size sufficiency in interview‐based studies: systematic analysis of qualitative health research over a 15‐year period. BMC Med Res Methodol. 2018;18(1):148. 10.1186/s12874-018-0594-7 30463515 PMC6249736

[26] Scientific Software Development GmbH . ATLAS.ti; 2021. Accessed March 25th, 2021. https://atlasti.com/

[27] Fetters MD , Curry LA , Creswell JW . Achieving integration in mixed methods designs—principles and practices. Health Serv Res. 2013;48(6pt2):2134‐2156. 24279835 10.1111/1475-6773.12117 PMC4097839

[28] Ministerio de Salud (MINSA) . Directiva Sanitaria 110‐MINSA/2020/DGIESP Lima. MINSA; 2021.

[29] Ministerio de Salud (MINSA) . Directiva Sanitaria N° 111‐MINSA/2020/DGOS Lima. MINSA; 2020.

[30] Fronteira I , Sidat M , Magalhães JP , et al. The SARS‐CoV‐2 pandemic: a syndemic perspective. One health. 2021;12:100228. 10.1016/j.onehlt.2021.100228 33614885 PMC7887445

[31] Organization WH . The Impact of the COVID‐19 Pandemic on Noncommunicable Disease Resources and Services: Results of a Rapid Assessment; 2020.

[32] Lion KC , Brown JC , Ebel BE , et al. Effect of telephone vs video interpretation on parent comprehension, communication, and utilization in the pediatric emergency department: a randomized clinical trial. JAMA Pediatr. 2015;169(12):1117‐1125. 26501862 10.1001/jamapediatrics.2015.2630 PMC5524209

[33] Clary L , Wang C , Byrne ME , Monaghan M . COVID‐19 pandemic‐related practices and policies affecting the continuity of behavioral health care among children with diabetes. Transl Behav. Med. 2020;10(4):819‐826. 32710626 10.1093/tbm/ibaa072 PMC7529096

[34] American Association of Diabetes Educators . An effective model of diabetes care and education: revising the AADE7 self‐care Behaviors(®). Diabetes Educ. 2020;46(2):139‐160. 31928334 10.1177/0145721719894903

[35] Elbarbary NS , Dos Santos TJ , deBeaufort C , Agwu JC , Calliari LE , Scaramuzza AE . COVID‐19 outbreak and pediatric diabetes: perceptions of health care professionals worldwide. Pediatr Diabetes. 2020;21(7):1083‐1092. 32686287 10.1111/pedi.13084 PMC7404589

[36] Scott SN , Fontana FY , Züger T , Laimer M , Stettler C . Use and perception of telemedicine in people with type 1 diabetes during the COVID‐19 pandemic—results of a global survey. Endocrinol. Diabetes Metab. 2020:e00180. 10.1002/edm2.180 33532617 PMC7831200

[37] Andrikopoulos S , Johnson G . The Australian response to the COVID‐19 pandemic and diabetes–Lessons learned. Diabetes Res Clin Pract. 2020;165:108246. 10.1016/j.diabres.2020.108246 32502693 PMC7266597

[38] Jethwani P , Saboo B , Jethwani L , et al. Management of children and adolescents having type 1 diabetes during COVID‐19 pandemic in India: challenges and solutions. Int J Diabetes Dev Ctries. 2020;40(3):335‐339. 32952333 10.1007/s13410-020-00865-w PMC7490475

[39] Pancheva V . Patient organizations an important unit in diabetes care. Knowl Int J. 2018;28(2):575‐580.

[40] Superintendencia Nacional de Salud (SUSALUD) . Afiliados por distrito; 2021. http://datos.susalud.gob.pe/dataset/consulta‐afiliacion/resource/7238ac1f‐7a69‐40c4‐ae84‐d01c46e95fc0

