# Supplementary material for: COVID‐19's impact on type 1 diabetes management: A mixed‐methods study exploring the Peruvian experience
Source: Int J Health Plann Manage. 2022 Jul 5:10.1002/hpm.3536. Online ahead of print. doi: 10.1002/hpm.3536 (PMC9349690; doi:10.1002/hpm.3536)
Supplement: Supplementary file 1 — Supplementary Material [file HPM-9999-0-s002.docx]

### Supplementary Table 1. Characteristics of participants that were interviewed

| Participants | Ubication | Health insurance | Number |
| --- | --- | --- | --- |
| T1DM or caregiver | Patient association representatives |  | 3 |
|  | Lima (capital city) | MINSA | 2 |
|  |  | EsSalud | 2 |
|  |  | Private | 2 |
|  | Province | MINSA | 4 |
|  |  | EsSalud | 5 |
|  |  | Private | 1 |
| Health care professionals (physicians, nurses, nutritionists) | Lima (capital city) | MINSA | 3 |
|  |  | EsSalud | 2 |
|  |  | Private | 1 |
|  | Province | MINSA | 1 |
|  |  | EsSalud | 1 |
|  |  | Private | 1 |
| Health care professionals (pharmaceutics) | Lima | MINSA | 2 |
|  |  | Private | 1 |
|  | Province | MINSA | 1 |

MINSA: Ministerio de Salud, EsSalud: Seguro Social de Salud

## Supplementary table 2. Reacting to the emergency and the system’s informal response theme

| Subtheme | Supporting data – qualitative study |
| --- | --- |
| Access to clinical visits | During the emergency state declared by the government, 56.5% (65/115) of the patients referred to have sought medical care. Of them, 50.8% (33/65) did not have access to medical consultations.  The main reasons for not seeking medical consultations were fear (30/50), considering it was not needed (9/50), hospitals not providing care for chronic patients’ (9/50) and lack of money (4/50), among other reasons, though it was risky (3/50), had difficulties related to transportation (2/50) and lose their job and insurance (1/50). |
| Reduction of resources for T1DM | 68.5% (37/54) of the health professionals interviewed reported that the capacity to care for patients with diabetes in their establishment had been reduced by at least 60%.  *“respondent: …The priority was to cover other areas… for example, 50% of pediatric emergency doctors were not there (because they were on leave). So, (those positions) had to be filled anyway, and also, differentiated services were created.*  *interviewer: How so?*  *respondent: In the case of pediatrics, there were two areas of hospitalization (…) one COVID and one non-COVID. There were no new doctors hired. So, it was the same group of people that had to work (on both areas)”.* (Provider_EsSalud_Lima) |
| Budgetary constraints | *“respondent: …I had hired an endocrinologist, but his contract was not extended to hire staff for COVID. In other words, his post was lost. As a patient with diabetes, instead of strengthening (the population with diabetes) because it is a vulnerable population, I believe that (…) this has made them more vulnerable*…” (Provider_MoH_Region) |
| Alternative communication strategies | *“respondent: (quarantine initiated between 15^th^ and 16^th^ march) There was a time when we were not sure how long it would be. We did not know. So, we waited a week. We saw that this situation (…) would last for a long time. So, we decided to attend (patient consultations) via WhatsApp. We coordinated with the Director of the hospital, with the pharmacy, with the SIS and we began to attend…”* (Provider_MoH_Region) |
| Shift from face-to-face to remote consultations | Before COVID-19, 46.2% (6/13) of the physicians had less than 10 face-to-face consultations per month and 46.2% (6/13) had more than 21. 92.3% (24/26) conducted less than 10 remote consultations per month.  After COVID-19, 46.2% (6/13) of the physicians had less than 10 face-to-face consultations per month, 38.5% (5/13) had between 11 to 20, and 15.4 (2/13) had more than 21. As for remote consultations, 26.9% (7/26) had less than 10 consultations per month, 23.1% (6/26) had between 11 to 20 and 50% (13/26) had more than 21 consultations per month.  *“respondent: Of the consultations that we usually had in the hospital, which could be 2000 consultations per month, we did 100. There was no type of attention (at the beginning of the pandemic). After June, as of July, (…), the hospital began to organize for outpatient consultation, but, above all, diabetic foot. What was organized was consultations by telephone (teleconsultations)”* (Provider_MoH_Lima) |

## Supplementary table 3. System’s formal response theme

| Subtheme | Supporting information |
| --- | --- |
| Active follow-up of patients | *“respondent: …50% was left without care because one group did not use medication. Then, we called and ask patients if they had medicines. One group did not answer the phone, another one had already changed phones. That kind of things. A small group just didn't have a phone number"* (Providers_EsSalud_Lima) |
| Difficulties with telemedicine – human resources | *“respondent: …the nurses who are working as receptionists right now are old ladies, (they do it) since they are in quarantine. Sometimes they provide references wrongly. I get calls for psychiatry that needed nutrition. (...) the patients are lost because they no longer call…” (*Provider_MoH_Lima*)* |
| Limitations of remote consultations | *“respondent: …teleconsultations imply losing some elements, such as the patient's triage (…) we cannot assess the patient's weight and height directly, or have immediate glucose at that time. (…) eye contact is also lost. Sometimes the patients come with food to the consultation, we assess that. A patient with diabetes brings unsuitable food. Another issue is the participation of the caregiver. Through the phone, we talk more with the parents and very seldom we interact with the child directly…”* (Provider_EsSalud_Region) |
| Suggestions to improve remote consultations | *“respondent: … a better differentiation should be made (in care), especially for children and young people with type 1 diabetes. They are very vulnerable to severe acute complications due to the interruption of their treatment or serious chronic complications also if medium and long-term follow-ups are not done. If medium or long-term follow-up is not done, complications of type 1 diabetes will be more frequent, the microvascular ones. So, specific measures need to be taken to provide greater attention to patients with type 1 diabetes…”* (Provider_EsSalud_Region)  *“respondent: Look, I have always thought that when you have a good registry of patients, data, numbers, cell phones and if the government or the type 1 diabetes program were interested in following up patients ... well, the first thing they would have to do is to have good patient data”* (Provider_MoH_Lima) |

## Supplementary table 4. Access to insulin theme

| Subtheme | Supporting information |
| --- | --- |
| Remote consultations and insulin prescription | *“respondent: …Telemonitoring is for continuators. That means that if I know the patient, I simply continue with the prescription. Everything by virtual means (…) they gave me a list of my patients, I make the phone call, we see what they need and I put to enter the prescription in my machine. The next day the pharmacy gives the patient the prescription with their products."* (Providers_MoH_Region) |
| Responses to the need for insulin - patients | *"respondent: When the pandemic started, I had the exact amount of insulin and we were (…) taking the food out of their mouth so they don't eat too much and we saved insulin."* (Patient_MoH_Region)  *“respondent: …I had to go looking in all of Chiclayo and its surroundings. Thank God I got (insulin) in a pharmacy, not the one I needed, but the rapid insulin (…) Well, since my son had already used it when he was in a diabetic coma, in the ICU, the doctor indicated that the rapid could be used, but in smaller doses.*  *interviewer: Did you somehow make up for it?*  *respondent: Exactly, exactly, because my son could not be without his insulin”* (Patient_EsSalud_Region) |
| Responses to the need for insulin – health care providers | Around half (24/44) of the professionals referred to having changed the insulin prescription of their patients due to the following motives: availability (21), and costs (8), among others. |
| Responses to the need for insulin – patient associations | *“respondent: …when we were in almost total confinement, we couldn't go to the pharmacies. We called for them to send us the insulin, to send us the strips and there were none, there were none. So, what we do in the chat ‘please, who has a (specific type of insulin), could you lend it to this person and then, when his insurance gives him one, she will return it’, we have seen ourselves in that obligation. You know that I have 50 strips extra, I donate them to the ones in need and so on. In other words, somehow making up for the deficiencies of the State and even private insurers”.* (Association 3) |
| Patient’s income | People living with T1DM and caregivers were also impacted economically because of the different measures implemented during the sanitary emergency. With regards to income, 91.5% (108/118) referred their monthly income had been reduced during the pandemic, while 50.9% (60/118) referred that their monthly income was lower than the national minimum wage. Which reduced their capacity to access insulin and devices for the management of T1DM. |

### Supplementary table 5. Summary of the system’s response of patients with T1DM needs

| Needs | Restriction/problem | Systems and patients’ response | Barriers and lessons learned |
| --- | --- | --- | --- |
| Continuity of care | Difficulties to mobilize to health establishment, need to minimize exposure to COVID-19, fear  Reduction of outpatient services (external consultation and laboratory testing).  Patients could not access the periodical controls (for example, glycated hemoglobin) programmed during the pandemic | The MoH implemented teleconsultations to reduce exposure of patients with chronic conditions to COVID-19. | There was not an adequate infrastructure nor trained health care professional.  There are not updated registries to track patients.  Even when laboratory testing was indicated, patients could not access them given the restriction (closure of outpatient services) and fear of COVID. Suggestion to decentralize laboratory testing by implementing laboratories in different regions and levels of care or collecting samples at the primary level to be processed at higher levels of care.  Health care professionals and patient found teleconsultations useful. It could be used as a complement of face-to-face consultations after COVID to facilitate more frequent consultations.  There is a need to decentralize care. |
| Access to insulin | Human insulins are covered by health care insurances. In some cases, analog insulins are also covered.  Teleconsultations facilitated the prescription of insulin. However, availability and difficulties to mobilize to health care establishments to pick up insulin were a limitation. | The MoH, Social Security and private health establishments proposed home delivery of drugs (or pick-up in the closest pharmacy).  As for the availability of insulin, pharmacies coordinated the transfer of drugs from over- to under-stocked establishments.  Patients and caregiver’s responses to the need for insulin consisted of reducing the dose of insulin, replacing the insulin they were using with another one available or cheaper, coordinate with patient associations, family and friends to help get and deliver the insulin, and buying insulin.  The median expenditure in insulin the month before the pandemic was 200 PEN (IQR: 150 a 300) and 250 PEN (IQR: 150 a 360) during the pandemic. No statistical differences were found before and after expenditure in insulin and, when interviewed, patients mentioned that prices remained stable.  Health care providers’ response to the lack of availability of insulin included changing the prescription and coordinating the transfer of insulin from public pharmacies with low usage of insulin to those with higher usage of insulin. | From the interviewed patients, only those with private insurance benefited from delivery.  Centralize care (in Lima), presented as a barrier as patients who received prescriptions from a hospital in Lima, but were living in other regions had to send a family member to pick up the insulin and then mailed it.  Patient organizations responded to provide support to patients who needed insulin.  Many patients with T1DM usually buy insulins. The prices remained stable during the pandemic. |
| Insulin syringes | In the case of syringes, they are covered by health insurances but are not always available so patients usually buy them. | No specific interventions were implemented to improve patients’ access to insulin syringes.  Patients had to buy them. The median expenditure in the month before the pandemic on syringes was 100 PEN (IQR: 50 to 140). This expenditure was not statistically different from the expenditure during the pandemic. However, some patients mentioned a small increase in the prices. | N/A |
| Monitoring devices (glucometers and test strips) | Access to test strips is usually variable, but most establishments do not provide them to patients. Thus, patients with T1DM usually buy test strips and glucometers and, during the pandemic, they continued purchasing these supplies. | No specific interventions were implemented to improve patients’ access to monitoring devices.  Given the difficulties to get appointments and prescriptions, some patients who usually received the strips, had to purchase them. Which in turn increased the monthly expenditure on these supplies. Thus, the median expenditure in test strips the month before the pandemic was 150 PEN (IQR: 100 to 250) and 200 PEN (IQR: 100 a 300) during the pandemic (p-value < 0.05).  Patients also opted to reduce the number of controls they usually conducted per day to spare strips and manage till the end of the month. | N/A |
| Other | Lockdown prevented people from going out to exercise (within the first months of the pandemic).  As for nutrition, mobility restrictions caused a lack of availability and increase prices of fruits and vegetables.  Patients felt abandoned and afraid of getting infected as media informed that patients with diabetes were considered a high-risk population. | No specific intervention was implemented with regards to physical activity, nutrition and mental health. | Mental health is an important component that was neglected in the system’s response to continuing chronic conditions care. |

N/A not applicable
