# Supplementary material for: COVID‐19's impact on type 1 diabetes management: A mixed‐methods study exploring the Peruvian experience
Source: Int J Health Plann Manage. 2022 Jul 5:10.1002/hpm.3536. Online ahead of print. doi: 10.1002/hpm.3536 (PMC9349690; doi:10.1002/hpm.3536)
Supplement: Supplementary file 2 — Supplementary Material [file HPM-9999-0-s001.docx]

# Online material: online surveys and interview guides

## Encuestas virtuales

Encuesta para personas con diabetes tipo 1 o cuidadores

Borrador de encuesta: <http://redcap.upch.pe/redcap/surveys/?s=XL7P9P44D9>

**Datos preliminares (criterios de inclusión)**

1. ¿Tiene usted diabetes tipo 1? Si/no
2. ¿Es usted cuidador de una persona con diabetes tipo 1? Si/No
3. ¿Vive usted en Perú? Si/No

**Datos demográficos de la persona con DMT1**

1. Edad:
2. Sexo:
3. Región en la que vive:
4. Provincia en la que vive:
5. ¿Cuenta con un seguro de salud? Si/No
   1. Si cuenta con seguro: ¿Con qué seguro cuenta? SIS, EsSalud, Privado, Fuerzas armadas
6. ¿Pertenece a alguna asociación de personas con diabetes o grupo de apoyo? Si/No
   1. Si pertenece a asociación: ¿A qué asociación pertenece? ADINA, D1 Perú, ADJ, otro

**Manejo de su diabetes tipo 1 habitualmente**

1. ¿Cuánto tiempo vive con DMT1?
2. Habitualmente, ¿Cuántas consultas tiene al año para control de DMT1?
3. ¿Qué tipo de insulina utiliza? (desplegable)
4. ¿Utiliza glucómetro y tiras reactivas para monitorear su glucosa? Si/No
   1. Si utiliza: ¿Cuántas tiras reactivas utiliza, en promedio, semanalmente?
5. ¿Utiliza algún dispositivo de monitoreo de glucosa continuo o intermitente? Si/No
   1. Si utiliza un dispositivo: ¿Qué dispositivo utiliza?
6. ¿Utiliza bomba de insulina? Si/No
7. ¿Usted realiza algún gasto para comprar insulina? Si/No
   1. Habitualmente, ¿Cuánto dinero gasta en promedio por mes en insulinas?
8. ¿Usted realiza algún gasto para comprar jeringas de insulina? Si/No
   1. Habitualmente, ¿Cuánto dinero gasta en promedio por mes en jeringas de insulina?
9. ¿Usted realiza algún gasto para comprar tiras reactivas? Si/No
   1. Habitualmente, ¿Cuánto dinero gasta en promedio por mes en tiras reactivas?
10. ¿Usted realiza algún gasto para comprar otros dispositivos de monitoreo (por ejemplo, FLASH o dispositivos de monitoreo continuo)? Si/No
    1. Habitualmente, ¿Cuánto dinero gasta en promedio por mes en otros dispositivos de monitoreo?

**Impacto del estado de emergencia**

1. ¿Sus ingresos familiares se han visto afectados durante el estado de emergencia? Si/No
2. ¿Su ingreso económico mensual familiar durante el estado de emergencia ha sido menor a 930 soles? Si/No

**Acceso a servicios de salud durante el estado de emergencia**

1. ¿Ha buscado atención médica por DMT1 durante el estado de emergencia? Si/No
   1. Si la respuesta si pasar a la pregunta siguiente
   2. Si la respuesta es no -> ¿por qué?: Miedo, dificultades de transporte, otros
2. ¿Ha podido acceder a consultas médicas por la DMT1 o alguna de sus complicaciones? Si/No
   1. Si la respuesta es sí: Esta consulta fue: presencial, telemedicina (llamada por teléfono o video llamada)
3. ¿Su médico tratante/personal de salud se comunicó con usted para hacer un seguimiento de su diabetes? Si/No

**Acceso a medicamentos (compra y costos)**

1. ¿Ha podido adquirir de forma gratuita insulina a través de su seguro? Todo/la receta parcial/no
2. ¿Ha comprado insulina? Si/No
   1. ¿Qué insulina ha comprado? (desplegable)
   2. ¿Cuánto ha gastado en promedio al mes de insulina?
   3. ¿Cuántas farmacias tuvo que visitar hasta que encontró la insulina?
   4. ¿En qué tipo de farmacia compró?: pública o privada
3. ¿Ha podido adquirir de forma gratuita jeringas para insulina a través de su seguro? Todo/ una parte de lo que necesitaba /no
4. ¿Ha comprado jeringas para insulina? Si/No
   1. ¿Cuánto ha gastado en promedio por jeringa de insulina?
   2. ¿Cuántas farmacias tuvo que visitar hasta que encontró las jeringas de insulina?
   3. ¿En qué tipo de farmacia compró?: pública o privada
5. ¿Ha podido adquirir de forma gratuita tiras reactivas a través de su seguro? Todo/una parte de lo que necesitaba/no
6. ¿Ha comprado tiras reactivas? Si/No
   1. ¿Cuánto ha gastado en promedio por tira reactiva?
   2. ¿Cuántas farmacias tuvo que visitar hasta que encontró las tiras reactivas?
   3. ¿En qué tipo de farmacia compró?: pública o privada

**Manejo de diabetes durante el estado de emergencia**

|  | El mes anterior a la cuarentena | Durante la cuarentena |
| --- | --- | --- |
| 1. ¿Ha tenido días sin tratamiento con insulina? | Si/no | Si/no |
| 1. ¿Ha tenido que racionar su dosis de insulina para que alcance? | Si/no | Si/no |
| 1. ¿Ha acudido a emergencia por alguna complicación de su diabetes? | Si/no | Si/no |

34. ¿Ha cambiado de insulina durante el estado de emergencia? Si/No
Si la respuesta es sí, preguntar -> ¿por qué?

- Costos
- Disponibilidad
- Otros

35. ¿Ha tenido dificultades para mantener sus cuidados nutricionales? Si/No
Si la respuesta es sí, preguntar -> ¿por qué?

- Costos
- Dificultad para conseguir alimentos
- Otros

36. ¿Ha tenido dificultades para mantener su actividad física? Si/No

Encuesta para profesionales de salud (médicos/enfermeras/educadores en diabetes)

Borrador de encuesta: <http://redcap.upch.pe/redcap/surveys/?s=KA9MXWAJ8C>

**Datos sociodemográficos**

Género:

- Femenino
- Masculino
- Otro

¿Qué tipo de personal de salud es Ud.?

- Médico/a general
- Médico/a de familia
- Endocrinólogo/a
- Endocrinólogo/a pediatra
- Pediatra
- Enfermero/a
- Nutricionista

¿Es usted educador en diabetes? Si/No

¿En qué región trabaja actualmente?: colocar un desplegable con todas las regiones

¿En qué provincia trabaja actualmente?: colocar un desplegable con las provincias por región

Ud. Es personal de salud de cuál de los siguientes subsistemas de salud (Si trabajara en más de uno, marque el subsistema al que le dedica más horas):

- MINSA
- EsSalud
- Fuerzas Armadas
- Privado

Si no es privado se abrirá la siguiente pregunta: ¿en qué nivel trabaja?

- Primario (postas y centros de salud)
- Secundario (hospital sin especialista)
- Terciario (hospital con especialista)

¿Ud. ha atendido pacientes con diabetes desde el inicio de la cuarentena?

- Si
- No

¿Ud. ha atendido pacientes con diabetes tipo 1 desde el inicio de la cuarentena?

- Si
- No

¿Los pacientes con diabetes tipo 1 fueron atendidos en (puede marcar más de uno)?

- Consulta externa
- Emergencia
- Hospitalización
- Teleconsulta

¿Puede dar un número promedio al mes de pacientes con diabetes tipo 1 a los que usted atiende/atendió en emergencia antes y después del estado de emergencia?

|  | Antes | Después |
| --- | --- | --- |
| Menos de 5 |  |  |
| Entre 5 y 10 |  |  |
| Entre 11 y 20 |  |  |
| Entre 21 y 30 |  |  |
| Más de 30 |  |  |

¿Puede dar un número de promedio al mes de pacientes con diabetes tipo 1 a los que usted atiende/atendió en hospitalización antes y después del estado de emergencia?

|  | Antes | Después |
| --- | --- | --- |
| Menos de 5 |  |  |
| Entre 5 y 10 |  |  |
| Entre 11 y 20 |  |  |
| Entre 21 y 30 |  |  |
| Más de 30 |  |  |

¿Puede dar un número de promedio al mes de pacientes con diabetes tipo 1 a los que usted atiende/atendió en consulta externa antes y después del estado de emergencia?

|  | Antes | Después |
| --- | --- | --- |
| Menos de 5 |  |  |
| Entre 5 y 10 |  |  |
| Entre 11 y 20 |  |  |
| Entre 21 y 30 |  |  |
| Más de 30 |  |  |

¿Puede dar un número de promedio al mes de pacientes con diabetes tipo 1 a los que usted atiende/atendió en teleconsulta antes y después del estado de emergencia?

|  | Antes | Después |
| --- | --- | --- |
| Menos de 5 |  |  |
| Entre 5 y 10 |  |  |
| Entre 11 y 20 |  |  |
| Entre 21 y 30 |  |  |
| Más de 30 |  |  |

¿Han tenido que modificar el tipo de insulina que prescriben a sus pacientes por motivos no clínicos? Si/No

Si la respuesta es sí, preguntar -> ¿por qué?

- Costos

- Disponibilidad

- Otros

Con respecto al establecimiento de salud en el que trabaja, ¿Se redujo la capacidad de la consulta externa en…?

-Menos de 10%

-Entre 10 a 30%

-Entre 30 a 60%

-Entre 60 a 90%

- Más de 90%

Encuesta para farmacéuticos

Borrador de encuesta: <http://redcap.upch.pe/redcap/surveys/?s=LNT9K33AJN>

**Datos sociodemográficos**

1. Género: Femenino / Masculino
2. Edad
3. Tiempo desde que labora en el establecimiento

**Datos del establecimiento**

1. Región
2. Provincia
3. Tipo subsistema: MINSA/ EsSalud / Fuerzas Armadas / Privados
4. Nivel de atención: Primer nivel / Segundo nivel / Tercer nivel

**Atención de pacientes con diabetes**

1. ¿Se ha atendido recetas de medicamentos para el tratamiento de diabetes? Sí/ No
2. De los medicamentos para el tratamiento de diabetes se han atendido recetas para pacientes con: Diabetes tipo 1/ Diabetes tipo 2 / Ambos
3. La atención de recetas ha sido mediante: (puede marcar más de una opción)

- Dispensación en farmacia de consultorio externo
- Dispensación en farmacia de emergencia
- Para servicio por delivery
- Por programas (ej. PADOMI)

**Dispensación de insulinas**

1. Durante el estado de emergencia ¿Se ha dispensado algún tipo de insulina? Sí/ No

- Si la respuesta es SÍ 🡪 ¿Qué tipo?
- Si la respuesta es No -> siguiente

1. Durante el estado de emergencia ¿Se ha recepcionado en almacén algún tipo de insulina? Sí/ No

- Si la respuesta es SÍ 🡪 ¿Qué tipo?
- Si la respuesta es No -> siguiente

1. Durante el estado de emergencia ¿Se ha agotado el stock de algún tipo de insulina? Sí/ No

- Si la respuesta es SÍ 🡪 ¿Qué tipo?
- Si la respuesta es No -> siguiente

1. ¿Puede dar un número promedio de atenciones por mes en que se dispensa insulinas en un mes normal?

- Menos de 5
- Entre 5 y 10
- Entre 11 y 20
- Entre 21 y 30
- Más de 30

1. ¿Puede dar un número promedio de atenciones por mes en que se ha dispensado de insulinas durante el estado de emergencia?

- Menos de 5
- Entre 5 y 10
- Entre 11 y 20
- Entre 21 y 30
- Más de 30

**Precio y disponibilidad de insulinas**

1. ¿Cuenta con algún tipo de insulina en el establecimiento? Sí/ No

- Si la respuesta es SÍ 🡪 desplegable (tipo de insulina, nombre, concentración, laboratorio) – llenar el precio

1. En el establecimiento ¿Cuál es la cobertura para dispensación de insulinas? (puede marcar más de una)

- Cubierta por el seguro de salud
- Mediante pago completo
- Mediante co-pago

1. ¿Cuenta con tiras reactivas?

- Si -> ¿las dispensa?
- No -> ahí queda

1. ¿Cuenta con jeringas para insulina (NO tuberculina)?

- Si -> ¿las dispensa?
- No -> ahí queda

## Guías de entrevista

**Evaluación del impacto de la pandemia de COVID-19 en el acceso a servicios de salud de las personas con Diabetes Mellitus Tipo 1**

**GUÍA DE ENTREVISTA PARA PACIENTES Y CUIDADORES**

Hola, mi nombre es ______________________, y el día de hoy le haré la entrevista. Para proteger su identidad, usaremos un seudónimo en nuestros informes y publicaciones. ¿Qué nombre o seudónimo desea que usemos durante esta? ____________________________

Antes de iniciar la entrevista, confirmar que el participante ha leído el consentimiento informado y comprende los objetivos del estudio y desea participar de forma voluntaria.

Confirmar los datos sociodemográficos brindados en la encuesta virtual y proceder con la entrevista.

1. ¿A qué edad le diagnosticaron la Diabetes Tipo1?
2. ¿Con qué médico u establecimiento de salud se suele controlar?
3. Durante la cuarentena ¿Ha acudido a un centro de atención distinto al que utiliza usualmente? ¿Por qué?
4. ¿Ha intentado usted acceder a consultas médicas o teleconsulta para DMT1?
   1. SI LA RESPUESTA ES SI: ¿Cuénteme lo que hizo para acceder a una consulta? (Los pasos) ¿Cómo evalúa esta experiencia?
   2. SI LA RESPUESTA ES NO: En caso no haya intentado acceder a una consulta médica, ¿Por qué motivo no intentó acceder?
5. ¿Qué seguro tiene?
6. Cuando se inició el Estado de Emergencia, ¿su seguro hizo cambios para continuar brindándole la atención que requiere? Cuénteme.
7. ¿Ha logrado obtener sus medicamentos (insulina, jeringas para insulina, tiras reactivas) durante el estado de emergencia?
8. ¿Quién le hizo entrega de sus medicamentos? (Explorar, seguro, su propio dinero, etc)
   1. Si el seguro le entregó los medicamentos, ¿Cuál fue el proceso que siguió (recojo en farmacia, delivery, etc.)?
   2. Si tuvo que comprar medicamentos, ¿Fue sencillo encontrar sus medicamentos en las farmacias? Cuénteme ¿cuántas farmacias tuvo que visitar? ¿El precio fue similar al precio habitual?
   3. Con respecto a antes del estado de emergencia, ¿tuvo la misma facilidad-dificultad para hallar y comprar sus medicamentos?
9. ¿Cómo afectaron las medidas del estado de emergencia (cierre de fronteras, limitación para movilizarse en el distrito, en la ciudad y entre ciudades) sus cuidados de DMT1?
10. SI NO HUBIERA SALIDO ESTA EN LA RESPUESTA ANTERIOR INDAGAR POR ¿El estado de emergencia ha afectado su economía o la de su familia? ¿Cómo esto ha afectado el cuidado de su DMT1? ¿Aproximadamente cuando ha gasto en el cuidado de laDM1 por efecto de la cuarentena?
11. Durante el estado de emergencia, ¿ha tenido problemas para controlar su dieta o su actividad física? Cuénteme ¿por qué? ¿Cómo los ha superado?
12. A nivel emocional durante el estado de emergencia, ¿ha tenido alguna dificultad o necesidad de apoyo psicológico? Cuénteme ¿por qué? ¿Cómo los ha superado?
13. Hay algo más que le gustaría agregar sobre la experiencia de ser una persona con DIABETES TIPO 1 en una situación de pandemia
14. Durante el estado de emergencia, ¿Si requiere una atención de emergencia, como ha sido la atención de los profesionales?

**Evaluación del impacto de la pandemia de COVID-19 en el acceso a servicios de salud de las personas con Diabetes Mellitus Tipo 1**

**GUÍA DE ENTREVISTA PARA PROFESIONALES DE LA SALUD**

Hola, mi nombre es ______________________, y el día de hoy le haré la entrevista. Para proteger su identidad, usaremos un seudónimo en nuestros informes y publicaciones. ¿Qué nombre o seudónimo desea que usemos durante esta? ____________________________

Antes de iniciar la entrevista, confirmar que el participante ha leído el consentimiento informado y comprende los objetivos del estudio y desea participar de forma voluntaria.

Confirmar los datos sociodemográficos brindados en la encuesta virtual y proceder con la entrevista.

1. ¿Me podría contar por favor hace cuánto tiempo trabaja en esta institución? ¿Trabaja en alguna otra? ¿Cuál? ¿Hace cuánto tiempo?
2. ¿Nos puede explicar cómo [nombre de la institución] se han organizado para brindar atención a pacientes con diabetes mellitus tipo 1 durante el estado de emergencia)?

🡪 Si no lo mencionara, preguntar por:

- consultas médicas,
- entrega de prescripciones,
- recojo de medicamentos
- manejo en emergencia,
- uso de telemedicina, entre otros

Si el entrevistado menciona que se está usando telemedicina, preguntar:

- 1. ¿cómo accedieron los usuarios a este servicio? ¿considera que algunos tuvieron dificultades para acceder? ¿quiénes? ¿qué se hizo al respecto? ¿qué se podría hacer a futuro?
  2. ¿qué otra infraestructura / capacitación / recurso humano podría necesitar para hacer telemedicina?

1. Sobre su actividad laboral ¿Hubo cambios en sus turnos? Cuénteme por favor
2. ¿Estos cambios lo han beneficiado? ¿cómo? Explorar si es que, por el contrario, lo han limitado
3. En cuanto a los pacientes ¿Ha percibido que durante el estado de emergencia ha variado la cantidad de pacientes con diabetes tipo 1 en el servicio de emergencia? ¿A qué cree que se debe?
4. En este contexto ¿Cuáles considera que han sido/son las barreras más grandes para brindar atención a los pacientes con diabetes tipo 1?
5. ¿Qué acciones Ud. y/o su servicio han tomado para superar estas barreras?

🡪 Si no hubieran tomado medidas ¿qué se podría mejorar?

1. ¿Qué facilidades Ud. y/o su servicio han tenido para brindar atención a los pacientes con diabetes tipo 1?
2. ¿Ha recibido capacitación sobre el cambio de los procesos discutidos?
3. Como profesional de la salud ¿Qué piensa que se podría hacer para estar mejor preparados para continuar manejando e estos pacientes en el contexto de la pandemia?

**Evaluación del impacto de la pandemia de COVID-19 en el acceso a servicios de salud de las personas con Diabetes Mellitus Tipo 1**

**GUÍA DE ENTREVISTA PARA QUÍMICOS FARMACEUTICOS**

Hola, mi nombre es ______________________, y el día de hoy le haré la entrevista. ¿Qué nombre o seudónimo desea que usemos durante esta? ____________________________

Antes de iniciar la entrevista, confirmar que el participante ha leído el consentimiento informado y comprende los objetivos del estudio y desea participar de forma voluntaria.

Confirmar los datos sociodemográficos brindados en la encuesta virtual y proceder con la entrevista.

1. ¿Hace cuánto tiempo trabaja en esta farmacia?
2. En el contexto del estado de emergencia por el COVID-19, en general ¿Cómo se ha organizado el área de farmacia dentro de su establecimiento? En el caso específico de la atención de los pacientes de DM1 ¿Cómo se ha organizado?
3. ¿Ha habido cambios en la cantidad de farmacias que están funcionando? Cuénteme.
4. Respecto al personal que labora ¿Han cambiado los horarios/número de personal asignado? Cuénteme
5. ¿Ha percibido si ha disminuido o aumentado el número de atenciones en farmacia para pacientes de DM1? ¿A qué cree que se debe?
6. En cuanto a los medicamentos para diabetes ¿Las entregas programadas de medicamentos al almacén se han visto afectadas? ¿Cómo?
7. ¿Han cambiado los procesos para dispensación de medicamentos en el establecimiento? ¿Cómo han cambiado (extensión de receta, otros)? ¿Han tenido dificultades en este nuevo proceso? ¿Cuáles?
8. ¿Cómo se ha organizado la provisión de medicamentos dentro de su establecimiento (delivery, envío a farmacia local, recojo por familiares, recojo habitual, recojo por profesional de salud, etc.)? ¿Esto fue similar para insulina?
9. ¿Se ha tomado alguna consideración especial para la dispensación de insulinas? ¿cuál?
10. ¿Su centro de labores realizó alguna capacitación sobre el cambio de los procesos para la atención? ¿Ha participado de la capacitación? En caso, no se hayan organizado capacitaciones ¿Cómo se ha informado para la atención en la farmacia?
11. ¿Qué piensa que se debería mejorar, pensando en que la pandemia va a continuar, para mejorar la calidad del servicio de farmacia?

**Evaluación del impacto de la pandemia de COVID-19 en el acceso a servicios de salud de las personas con Diabetes Mellitus Tipo 1**

**GUÍA DE ENTREVISTA PARA ASOCIACIONES DE PACIENTES**

Hola, mi nombre es ______________________, y el día de hoy le haré la entrevista. Para proteger su identidad, usaremos un seudónimo en nuestros informes y publicaciones. ¿Qué nombre o seudónimo desea que usemos durante esta? ____________________________

Antes de iniciar la entrevista, confirmar que el participante ha leído el consentimiento informado y comprende los objetivos del estudio y desea participar de forma voluntaria.

1. Cuénteme un poco sobre la asociación que preside (¿cuándo se creó? ¿cuál es su misión? ¿quiénes son sus miembros? ¿cuántos asociados tienen?)
2. Previo al estado de emergencia, ¿en qué consistían las actividades de su asociación? ¿Con qué frecuencia se realizaban? ¿Cómo se realizaban? ¿Cómo difundían estas actividades?
3. ¿Cómo afectaron las medidas del estado de emergencia (cierre de fronteras, limitación para movilizarse en el distrito, en la ciudad y entre ciudades) las actividades de su asociación? ¿Ha realizado alguna actividad virtual con su asociación durante el estado de emergencia? De haberla realizado, ¿Cómo evalúa esta actividad?
4. Previo al estado de emergencia, ¿qué canales de comunicación utilizaban sus miembros para mantenerse en contacto o intercambiar información?
5. Durante el estado de emergencia, ¿se afectó la comunicación entre los miembros de su asociación? ¿qué pasó?
6. Durante el estado de emergencia, ¿cuáles diría que han sido las mayores dificultades que han tenido que afrontar los miembros de la asociación?
7. Durante el estado de emergencia, ¿ha recibido comunicaciones por parte de sus miembros solicitando apoyo para sus cuidados de DMT1? Cuénteme ¿en qué consistió el apoyo solicitado? ¿Tuvo facilidades/dificultades para conseguir los recursos para apoyar a los asociados, en comparación al período previo al estado de emergencia?
8. Hay algo más que le gustaría agregar sobre la experiencia de la asociación (o de sus miembros) en una situación de pandemia
